# Job satisfaction of maternity care providers in the Netherlands: Does working in or with a birth centre influence job satisfaction?

**DOI:** 10.18332/ejm/94279

**Published:** 2018-09-13

**Authors:** Therese A. Wiegers, Marieke A. Hermus, Corine J. Verhoeven, Marlies E. Rijnders, Karin M. van der Pal-de Bruin

**Affiliations:** 1 NIVEL (Netherlands Institute for Health Services Research), The Netherlands; 2 Department of Child Health, TNO, The Netherlands, Department of Obstetrics, Leiden University Medical Center, Leiden, The Netherlands, Midwifery Practice Verloskundigen Oosterhout, The Netherlands; 3 Department of Midwifery Science, AVAG/Amsterdam, Public Health Research Institute, VU University Medical Center, Amsterdam, Department of Obstetrics and Gynaecology Maxima Medical Centre, Veldhoven, The Netherlands; 4 Department of Child Health, Leiden, The Netherlands; 5 Department of Medical Statistics, Leiden University Medical Center, The Netherlands

**Keywords:** birth centres, maternity care workforce, survey research

## Abstract

**INTRODUCTION:**

In the Netherlands birth centres have recently become an alternative option as places where women with uncomplicated pregnancies can give birth. This article focusses on the job satisfaction of three groups of maternity care providers (community midwives, clinical care providers and maternity care assistants) working in or with a birth centre compared to those working only in a hospital or at home.

**METHODS:**

In 2015, an existing questionnaire was adapted and distributed to maternity care providers and 4073 responses were received. Using factor analyses, two composite measures were constructed, a Composite Job Satisfaction scale and an Assessment-of-Working-in-or-with-a-Birth-Centre scale. Differences between groups were tested with Student’s t-test and MANOVA with post hoc test and linear regression analyses.

**RESULTS:**

The overall score on the Composite Job Satisfaction scale did not differ between community midwives or clinical care providers working in or with a birth centre and those working in a different setting. For maternity care assistants there was a small but significantly higher score for those not working in a birth centre. Maternity care assistants’ overall job satisfaction score was higher than that of both other groups. In a linear regression analysis working or not working in or with a birth centre was related to the overall job satisfaction score, but repeated for the three professional groups separately, this relation was only found for maternity care assistants.

**CONCLUSIONS:**

Job satisfaction is generally high, but, except for maternity care assistants, not related to the setting (working or not working in or with a birth centre).

## INTRODUCTION

In the Netherlands three groups of medical professionals are responsible for providing maternity care: midwives, obstetricians, and some general practitioners. In addition to these groups, maternity care assistants (MCAs) and nurses assist during labour and birth, and also provide care and advice during the postpartum period. Most midwives are community midwives, they are independently practicing care providers in primary care. They provide the full range of maternity care: antenatal, natal and postnatal care, to healthy women with uncomplicated pregnancies and births. In case of an underlying pathology, threatening complications or a request for pharmacological pain relief, a woman will be referred to an obstetrician in a hospital. Women with uncomplicated pregnancies previously had two options where to give birth, at home or in a hospital, both assisted by their own community midwife. However, recently, birth centres have become an alternative option in a number of regions in the Netherlands. Most birth centres do not employ a full staff of care providers but have agreements with community midwives to bring their clients to give birth in the birth centre, and with hospitals to refer clients in need of specialist care. A birth centre in the Netherlands is primarily an alternative location, not an alternative form of care. The majority of birth centres are located in a hospital but separated from the obstetric department.

Job satisfaction of care providers is one of the aspects to be considered in measuring the success of organisational changes, like the introduction of birth centres, but recent studies on job satisfaction among maternity care providers are scarce. A qualitative study by Warmelink et al.^1^ showed that direct client contact, positive support and teamwork, as well as the ability to work independently and autonomously, led to higher levels of satisfaction among Dutch primarycare midwives. However, no studies are available about job satisfaction among other professions in maternity care in the Netherlands.

In the last few decades the number of home births in the Netherlands has decreased rapidly^2,3^, from about 25% in 2000 to 13% in 2015. One of the reasons is the increased number of referrals to secondary care before and during labour (from about 46% in 2000 to 58% in 2015)^2,3^. Another reason could be the renewed discussions about the safety of home births, following the EURO-Peristat publications about perinatal mortality^4^. Because of this, fewer women choose to give birth at home, leading to an increased number of births in the hospital, assisted by a community midwife (from about 12% in 2000 to 16% in 2015)^2,3^. Responding to the Peristat publications, a strategic review of maternity services^5^ was performed, leading to a changing maternity service provision in the Netherlands, with the emphasis shifting towards more integrated care^6^.

The growing number of birth centres parallels the decrease in home births and the discussions about integrated care. Before 2000 only a few birth centres, called maternity clinics, existed in the Netherlands, but in the last ten to fifteen years their number increased rapidly^7^. Because birth centres are relatively new and there is discussion about their role in the changing maternity care system in the Netherlands, the Dutch Birth Centre Study was initiated to evaluate the effects of birth centre care on quality of care, experiences of clients and caregivers, economic outcomes and implications for future implementation of birth centre care^8^. After formulating the definition of a birth centre in the Netherlands^7^, the study identified 23 birth centres that were operational in September 2013. A sub-study of this project, focussing on the experience of caregivers, is presented in this article.

The research questions for this sub-study are: ‘Is there a difference in job satisfaction of care providers working regularly or occasionally in or with a birth centre, compared to care providers working only in other settings.’ and ‘How do care providers working in or with a birth centre assess that workplace?’.

## METHODS

### Data collection

Early in 2015 a questionnaire was distributed online through professional organisations of all professionals in maternity care and through hospitals to community midwives, clinical midwives, gynaecologists/obstetricians, paediatricians, maternity care assistants and obstetric nurses. No selection was made, everyone was invited to respond. The questionnaire was available from February 2015 until April 2015. In the third week of March, a reminder email was sent to all midwifery practices and other contact persons, and further reminders were placed on the KNOV-website (KNOV: Royal Dutch Organization of Midwives), and in forums such as the hospital midwives’ group within the KNOV.

The design and planning of this cross-sectional study were presented to the Medical Ethics Committee of the UMCU (University Medical Centre Utrecht), which confirmed that an official ethical approval of this study was not required.

### Questionnaire development

In cooperation with two other maternity care related studies in the Netherlands, an existing questionnaire was adapted for use among care providers in maternity care. The questionnaire is based on a validated instrument^9^ and consists of 10 themes with a total of 81 questions. The themes were: 1) general background, 2) staffing and organisation, 3) job demands and tasks, 4) social support in the workplace and closeness, 5) cooperation, 6) arrangements and handover, 7) autonomy, 8) development opportunities, 9) financial assessment and satisfaction, and 10) influence of the job on the private life. Except for Theme 1, answers were on a 4-point scale ranging from ‘totally disagree’ to ‘totally agree’. The questions were irregularly positively and negatively formulated but all answers were coded with 1 (the most negative) and 4 (the most positive) response with regard to job satisfaction, resulting in a neutral value of 2.5.

Two of the three studies added questions to the questionnaire, specific for their own research topic. For the Dutch Birth Centre Study, 21 questions related to working in or with a birth centre were added. All care providers were asked whether or not they worked in or with a birth centre, regardless of the intensity of that work relation (on a regular basis or occasionally).

Experiences of working in or with a birth centre were measured on four themes with a total of 14 questions also with answers on a 4-point scale ranging from ‘totally disagree’ to ‘totally agree’. These themes were: organisation, cooperation, location, and working conditions. Again, the questions were irregularly positively and negatively formulated but all answers were coded with 1 (the most negative) and 4 (the most positive) response with regard to work experiences. Nine of these questions also provided the option to answer: ‘I don’t know’ or ‘not applicable’. Finally, care providers were asked to indicate on a 4-point scale, ranging from ‘not at all’ to ‘very much’, how much the local birth centre had a positive influence on their job satisfaction.

### Statistical analyses

Factor analyses were used to construct two composite measures, a Composite Job Satisfaction scale for all maternity care providers and an Assessment-of-Workingin-or-with-a-Birth-Centre scale for care providers working regularly or occasionally in or with a birth centre.

Three groups of care providers: maternity care assistants (MCA), community midwives (CoM) and clinical care providers (CCP) (clinical midwives, obstetricians, paediatricians and obstetric nurses), working regularly or occasionally in or with a birth centre were compared to those working only in other settings. The clinical care providers, although of different professional background (40.3% obstetrician, 17.4% clinical midwife, 21.1% obstetric nurse, 16.2% paediatrician, 5% other), were combined into one group, as working in a clinical setting is what differentiates them from both other groups of comparison, i.e. community midwives and maternity care assistants, who may assist a woman giving birth in a hospital, but are not part of the clinical setting.

Within the group of care providers working in or with a birth centre, comparisons were made between the three groups. Differences between groups were tested with Student’s t-test and MANOVA with post hoc test. A linear regression analysis was conducted for the total group of professionals and for the three groups separately with the Composite Job Satisfaction scale as dependent variable. A p-value < 0.05 was considered to be statistically significant. SPSS was used for the analyses.

## RESULTS

### Participants

In all, 4073 respondents completed the questionnaire. Of these, 224 respondents were excluded from the analyses: 49 not working as a maternity care provider; 56 with a profession other than community midwife (CoM), clinical care provider (CCP) or maternity care assistant (MCA); and 119 who did not fill in their profession. This resulted in a total of 3849 respondents of whom 1038 (27%) were regularly or occasionally working in or with one of the birth centres included in the Dutch Birth Centre Study. An overview of the respondent’s occupation is seen in Table 1. A response rate could not be calculated because it is unknown how many professionals received the invitation to fill in the questionnaire. However, the total study population, eligible for this study, consists of about 9000 MCA^10^, 2000 CoM^10^ and 4600 CCP (± 800 clinical midwives^11^, ± 800 Gyn/Obs^12^, ± 2800 O&G nurses^13^).

**Table 1:** Respondents by profession

	*Total number*	*Working in/with birth centre (BC) n (%)*	*Not working in/with birth centre (not BC) n (%)*
Community midwives (CoM)	406	154 (37.9)	252 (62.1)
Clinical care providers (CCP)	598	142 (23.7)	456 (76.3)
Maternity care assistants (MCA)	2845	742 (26.1)	2103 (73.9)
Total number	3849	1038 (27.0)	2811 (73.0)

We did ask the care providers working in or with one of the birth centres what percentage of their clients received birth centre care, in order to have an idea of their involvement with birth centres. For only a few in each group (2.3% MCA, 1.3% CoM, 2.9% CCP) all clients receive (some of their) care in a birth centre. For the majority (68% MCA, 61% CoM, 84% CCP), 50% or less of their clients receive care in a birth centre.

As seen in Table 2, average age differs between professions, with community midwives being younger than other care providers. Men are clearly the exception among community midwives and maternity care assistants, but less among clinical care providers. The majority of community midwives and about one in four clinical care providers are self-employed. Clinical care providers are the most experienced in maternity care, as well as in their current jobs, while community midwives work on average the most hours per week, about twice as many as maternity care assistants.

**Table 2:** Background of respondents

	*Community midwives (CoM)*		*Clinical care providers (CCP)*		*Maternity care assistants (MCA)*	
	*BC(N=154)*	*Not BC(N=252)*	*BC(N=142)*	*Not BC(N=456)*	*BC(N=742)*	*Not BC(N=2103)*
Average age (years)	39.2	37.3	46.5	46.4	47.4	47.1
Percentage female	99.4	98.7	83.1	77.8	99.7	99.3
Employment status (%)						
Employed	9.7	7.6	69.7	73.0	90.8	87.4
Self-employed	79.2	80.6	28.2	24.9	6.9	8.7
Locum	9.7	11.0	1.4	0.7	0.4	0.6
Other	1.3	0.8	0.7	1.4	1.9	3.3
Total work experience in maternity care (years)	13.8	12.3	18.2	17.2	14.9	16.1
Work experience in current job (years)	10.4	9.4	12.2	11.7	10.0	11.2
Working hours per week	42.9	44.0	37.8	39.2	22.7	22.3

### Comparison of job satisfaction between different groups of care providers

For the Composite Job Satisfaction scale initially 13 factors were identified, with Cronbach’s alpha ranging from 0.71 to 0.99. One factor, ‘social support from supervisor’, was excluded from the Composite Job Satisfaction scale because it was not applicable to the majority of community midwives and more than a quarter of the clinical care providers, because they are self-employed.

**Figure 1:**
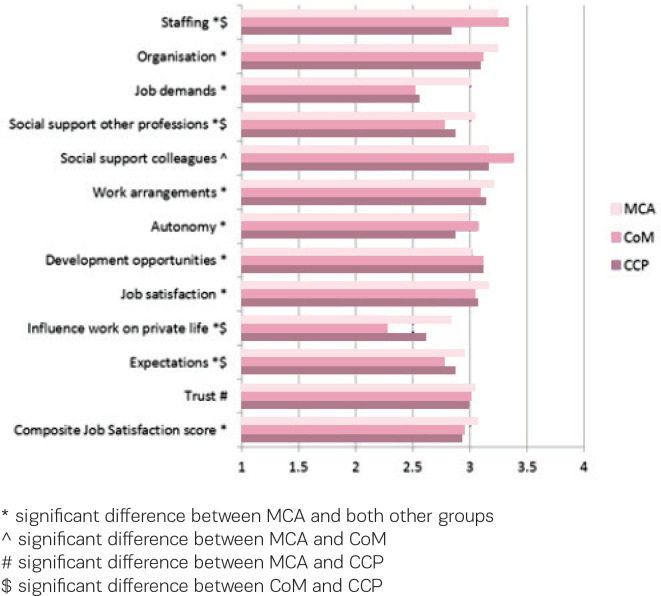
Factors included in the Composite Job Satisfaction scale (range 1–4) for the three groups of care providers: maternity care assistants (MCA), community midwives (CoM) and clinical care providers (CCP), with neutral value 2.5

Figure 1 shows the scores on the factors included in the Composite Job Satisfaction scale for the three different groups of care providers, regardless of their work setting. In general, maternity care assistants show higher levels of job satisfaction than the other care providers. Maternity care assistants score significantly different than both other groups on ten of the twelve factors and on the Composite Job Satisfaction scale. Only on the factor ‘social support from colleagues’ the difference with clinical care providers is not significant and on the factor ‘trust’ the difference with community midwives is not significant. Significant differences between community midwives and clinical care providers are found on the factors ‘staffing’, ‘social support other professions’, ‘influence work on private life’, and ‘expectations’.

### Comparison of job satisfaction between care providers working regularly or occasionally in or with a birth centre and care providers working only in other settings

**Figure 2:**
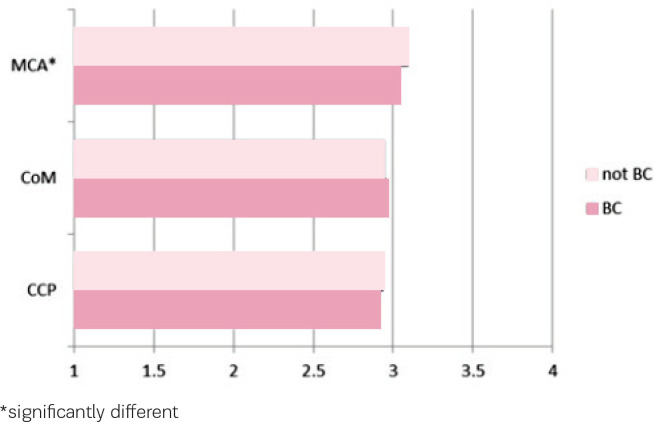
Composite Job Satisfaction scores of different groups of care providers: maternity care assistants (MCA), community midwives (CoM) and clinical care providers (CCP) regularly or occasionally working in or with a birth centre (BC) and care providers working only in other settings (not BC), range 1–4 with neutral value 2.5

Figure 2 shows the score on the Composite Job Satisfaction scale of different groups of care providers working regularly or occasionally in or with a birth centre, and care providers working only in other settings. The average score for the three groups of care providers working in or with a birth centre is 3.01, for the care providers working only in other settings the average score is 3.05. This difference is statistically significant (t=4.14, p<0.05), but looking at the different groups, this difference is only found among maternity care assistants. For community midwives and for clinical care providers there is no difference in the score on the Composite Job Satisfaction scale between both groups, but for maternity care assistants the score on the Composite Job Satisfaction scale is slightly higher for those not working in or with a birth centre (not BC) (3.09 vs 3.04, t=4.35, p<0.05).

Table 3 shows the scores on the factors included in the Composite Job Satisfaction scale for different groups of care providers by work setting. On individual factors some differences are found between the settings, but they are not tested, because of the many tests involved, with the risk of chance significances. However, one finding deserves mentioning, community midwives are the only ones to score lower than neutral: a score of 2.22 (BC) and 2.32 (not BC) on the factor ‘influence work on private life’, and a score of 2.46 for those working regularly or occasionally in or with a birth centre on the factor ‘job demands’. All other scores for all three groups of professionals are on the positive side of the scale, i.e. at or above 2.5.

**Table 3:** Job satisfaction of respondents working regularly or occasionally in or with a birth centre (BC) or working only in other settings (not BC), range 1–4 with neutral value 2.5

	*Community midwives (CoM)*			*Clinical care providers (CCP)*			*Maternity care assistants (MCA)*		
	*Total*	*BC*	*Not BC*	*Total*	*BC*	*Not BC*	*Total*	*BC*	*Not BC*
Staffing (3*)	3.34	3.35	3.32	2.83	2.81	2.84	3.25	3.19	3.26
Organisation (4)	3.11	3.08	3.12	3.10	3.12	3.09	3.23	3.21	3.24
Job demands (6)	2.51	2.46	2.54	2.55	2.50	2.57	3.01	2.97	3.02
Social support other professions (5)	2.75	2.76	2.73	2.86	2.90	2.85	3.04	3.00	3.05
Social support colleagues (5)	3.37	3.37	3.38	3.15	3.12	3.16	3.15	3.12	3.17
Work arrangements (4)	3.06	3.09	3.04	3.13	3.09	3.14	3.19	3.14	3.22
Autonomy (5)	3.07	3.09	3.05	2.85	2.80	2.87	2.98	2.96	2.99
Development opportunities (5)	3.11	3.17	3.06	3.12	3.09	3.13	3.01	3.00	3.02
Influence work on private life (3)	3.03	3.02	3.03	3.06	2.99	3.08	3.16	3.12	3.18
Working hours per week	2.28	2.22	2.32	2.61	2.58	2.62	2.84	2.82	2.86
Expectations (8)	2.76	2.76	2.76	2.88	2.87	2.88	2.96	2.93	2.98
Trust (6)	3.01	3.02	3.01	2.99	3.00	2.98	3.05	3.01	3.06
Composite Job Satisfaction score	2.95	2.95	2.95	2.93	2.91	2.94	3.07	3.04	3.09

* number of questions in the factor

Linear regression analysis was performed for the total group of professionals with the Composite Job Satisfaction scale as dependent variable and average age, profession, years of experience in their profession, years of experience in their current work setting, number of working hours per week, and working or not working in or with a birth centre as independent variables, using the backward method. As seen in Table 3, three of these six variables were related to the job satisfaction score: number of working hours per week (β=-0.038, 95% CI: -0.002–0.000), profession (β=-0.193, 95% CI: -0.088 – -0.057), and working or not working in or with a birth centre (β=-0.076, 95% CI: -0.066 – -0.024). When repeated for the three professional groups separately, for maternity care assistants only the variable working or not working in or with a birth centre was related to the job satisfaction score (β=-0.092, 95% CI: -0.079 – -0.029), with higher job satisfaction among maternity care assistants not regularly or occasionally working in or with a birth centre. For community midwives the only variable remaining was the years of experience in their profession, with higher job satisfaction related to fewer years of experience (β=-0.117, 95% CI: -0.007 – 0.000). For clinical care providers no single variable remained in the analysis.

### Assessment of working in or with a birth centre

For the Assessment-of-Working-in-or-with-a-Birth-Centre scale four factors were identified, with Cronbach’s alpha ranging from 0.63 to 0.88, but the number of valid responses for the composite scale was low, due to the large number of respondents, especially maternity care assistants (63%) and clinical care providers (70%), who indicated they did not know the answer to one or more of the questions.

**Figure 3:**
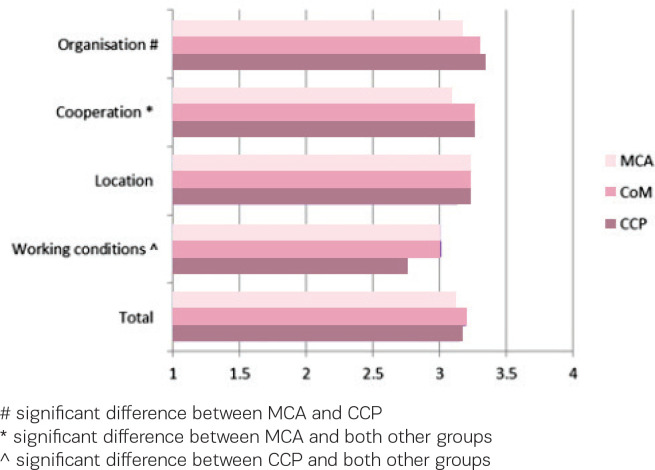
Assessment of working in or with a birth centre by maternity care assistants (MCA), community midwives (CM) and clinical care providers (CCP), range 1–4 with neutral value 2.5

Figure 3 shows the care providers’ assessment of working in or with a birth centre. All scores are on the positive side of the scale. In contrast to the scores on the Composite Job Satisfaction scale, maternity care assistants score on all four factors lower than both other groups of care providers. On the factor ‘organisation’, maternity care assistants score significantly lower than clinical care providers, on the factor ‘cooperation’ maternity care assistants score significantly lower than both community midwives and clinical care providers, while on ‘working conditions’ clinical care providers score significantly lower than both maternity care assistants and community midwives. On the factor ‘location’ and on the total score there are no differences between the groups.

**Figure 4:**
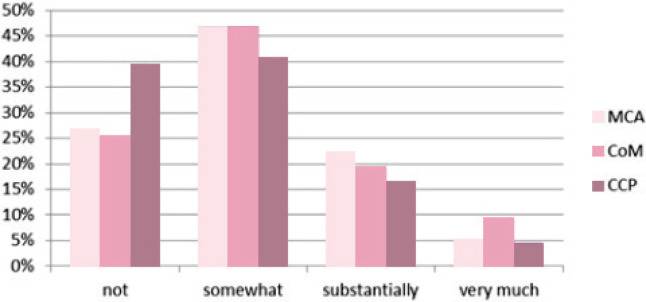
Answers of maternity care assistants (MCA), community midwives (CoM) and clinical care providers (CCP) to the question: ‘How much does the birth centre in your area influence your job satisfaction?’

Figure 4 shows that for 5 to 8% of care providers the birth centre has a large influence on their job satisfaction, while a minority in all three groups report that there is no influence. Whether this experienced influence is positive or negative is unclear, however.

### Comparison of job satisfaction between different birth centres

In the Dutch Birth Centre Study, 23 birth centres are included and clustered according to their location (freestanding, alongside or on-site), and to their integration profile (low, intermediate or high)^14^. The above presented data have also been checked for meaningful differences between these clusters, but there was none.

## DISCUSSION

Birth centres are a relatively new phenomenon in the Dutch maternity care system. For women with an uncomplicated pregnancy who do not want to give birth at home, birth centres provide an alternative to a hospital birth, with a home-like atmosphere, all kinds of conveniences, and their own midwife and a maternity care assistant to support them during labour and birth. In the Dutch Birth Centre Study several aspects of birth centre care were studied^4^, including client experiences^15^. In this sub-study we found that the overall score on the Composite Job Satisfaction scale is not different for community midwives and clinical care providers working regularly or occasionally in or with a birth centre compared to those working only in a different setting, while for maternity care assistants there was a small but significantly higher score for those not working in a birth centre. However, the maternity care assistants’ overall job satisfaction score was higher than the scores of both other groups. The overall finding is that for community midwives and clinical care providers the setting (the birth centre) does not significantly influence their job satisfaction but we can only guess why. Maybe there is not enough distinction between the birth centre and the maternity ward of the hospital, where lowrisk women can give birth assisted by their own midwife and where high-risk women receive specialist care. After all, most birth centres are located inside the hospital, on a different floor or even next to the obstetric ward.

We did find significant differences between community midwives and clinical care providers as groups, with higher scores for community midwives on the factor ‘staffing’, and lower scores on the factors ‘social support other professions’, ‘influence work on private life’ and ‘expectations’. We also found that community midwives score negative on the factor ‘influence of work on their private life’. This last finding has been found in other studies among midwives as well^1^, which is confirmed by the fact that it is found for both groups of midwives, those working regularly or occasionally in or with a birth centre and those working only in other settings.

Regarding the care providers’ assessment of working in or with a birth centre, we found that maternity care assistants were less positive than both other groups. The reason for this difference is not immediately clear but may be related to their limited experience with birth centres. Most maternity care assistants only work occasionally in a birth centre. Maternity care assistants work most of their time in private homes, providing care and support to families in the first week after the baby is born. Moreover, their involvement with childbirth, at home or in a hospital or birth centre, is only part of their job. Therefore it could be that they are or feel less involved in the organisation of a birth centre and the cooperation with other care providers.

Among the care providers working regularly or occasionally in or with a birth centre, clinical care providers are less positive about ‘working conditions’ than both community midwives and maternity care assistants. We have no explanation for this result, other than the fact that most clinical care providers are only indirectly involved with birth centres. Only when clients are referred to clinical care there will be contact between care providers in the birth centre and clinical care providers. Most of the time the referred clients will have to be transferred from the birth centre to the clinical department. Only in a few cases the clinical care providers will enter the birth centre to take over the care from the community midwife.

We found that maternity care providers are generally satisfied with the place they have chosen to work in. However, the different birth settings may be of more significance to the choices and experiences of couples having their baby than to the care providers assisting with childbirth. In addition, further research needs to address the job demands of midwives, as well as the influence of work on their private life.

This study has a number of limitations. First, there is no way of telling how selective our study population is, because we do not know how many and which professionals did not receive the invitation to fill out the questionnaire and who declined to respond. Secondly, it is possible that our questionnaire was not specific enough to discern a difference between care providers working in or with a birth centre and those working only in other settings. Moreover, every respondent who answered that they worked in or with a birth centre was included in that sub-group, regardless of the intensity of that work relation. However, as birth centres are still not common in the Netherlands, it is likely that midwives working in the vicinity of a birth centre will have had clients choosing to give birth there and will have experience with the birth centre. Thirdly, we did not differentiate between clinical care providers and included obstetricians, paediatricians, clinical midwives and nurses, because they all work in a clinical environment and within a clinical hierarchy, which is fundamentally different from the setting in a birth centre, where community midwives are independent care providers.

## CONCLUSIONS

Job satisfaction among maternity care providers is generally high, with only two of twelve factors resulting in a less than positive score among community midwives: ‘influence of work on their private life’ for both groups and ‘job demands’ for community midwives working in or with a birth centre. On the Composite Job Satisfaction scale no differences were found between community midwives and clinical care providers, while maternity care assistants score on average higher than both other groups. Only for maternity care assistants a difference is found between those working regularly or occasionally in or with a birth centre and those working only in other settings, with higher job satisfaction for the latter group. All three groups of care providers are positive about working in or with a birth centre and indicate that it influences their job satisfaction, but that influence is not visible in the overall Composite Job Satisfaction score.
